# Pandemic perspectives: the temporal influence of COVID-19 on attitudes toward marriage and childbirth

**DOI:** 10.3389/fpsyg.2025.1488014

**Published:** 2025-03-18

**Authors:** Jessica T. Campbell, Amanda N. Gesselman, Margaret Bennett-Brown

**Affiliations:** ^1^The Center for Evaluation, Policy, and Research, Indiana University, Bloomington, IN, United States; ^2^The Kinsey Institute, Indiana University, Bloomington, IN, United States; ^3^Department of Communications, Texas Tech University, Lubbock, TX, United States

**Keywords:** COVID-19, romantic relationships, attitudes, marriage, children, family

## Abstract

**Introduction:**

Marriage and childbirth rates in the U.S. have declined over the past decade, with individuals delaying these life milestones. The COVID-19 pandemic further complicated these trends, simultaneously increasing the desire for connection while disrupting relationship formation. This study examines how perceptions of marriage and childbearing shifted during the first two years of the pandemic using two cross-sectional, nationally representative surveys.

**Methods:**

Study 1 (*n* = 513), conducted in January 2021, and Study 2 (*n* = 1,850), conducted in August 2022, surveyed participants on the perceived importance of marriage and childbearing before and during the pandemic. Data were collected through online surveys and analyzed using linear regressions to identify demographic differences and shifts in attitudes.

**Results:**

Study 1 (*n* = 513) in January 2021 found that approximately 29% reported a decreased importance of marriage, while nearly 35% reported a reduced importance of child-bearing. Women and those with higher income reported an increase in the importance of marriage, while those with higher income reported an increase in the importance of having children. Study 2 (*n* = 1850) in August 2022 revealed a noteworthy contrast. Overall, participants expressed a consistent desire for marriage compared to pre-pandemic levels. Conversely, participants demonstrated a significant shift in their desire for children, indicating a greater inclination toward childlessness compared to pre-pandemic attitudes. 15.1% reported a reduced importance of having children. Higher income participants rated marriage as more important, while heterosexual participants rated having children as more important relative to pre-pandemic years.

**Discussion:**

These results underscore the dynamic nature of individuals’ perceptions surrounding marriage and childbearing in response to a transformative event like the COVID-19 pandemic. They provide valuable insights into the evolving cultural narrative around these milestones, highlighting the resilience and adaptability of societal values in the face of unprecedented change.

## 1 Introduction

Marriage and childbearing have traditionally stood as defining benchmarks of adulthood, symbolizing both societal and individual maturity (e.g., Coontz, 2005; Erikson, 1968). These rites of passage have historically been regarded as near-universal aspirations and held as societal expectations for proper adult development. Yet, recent decades have seen a pronounced shift in these cultural norms within the United States. This evolving landscape, characterized by an increasing propensity to delay marriage and having children, suggests a transformation in the valuation of these life events (Arnett, 2000; Payne, 2019; U.S. Census Bureau, 2021).

This gradual change was likely further compounded by the COVID-19 pandemic, which may have prompted a re-examination of personal priorities for many. The pandemic, with its inherent pressures and constraints, has brought about a number of challenges for individuals trying to navigate these traditional paths to adulthood. As such, and consistent with the cost–benefit analysis central to Social Exchange Theory (Homans, 1958; Thibault and Kelley, 1959), some individuals may perceive heightened risks and diminished rewards from the pursuit of marriage and parenthood. Moreover, Crisis Theory (Turner and Avison, 1992) suggests that the disarray wrought by an event like the COVID-19 pandemic could catalyze a search for deeper meaning, potentially recalibrating the personal significance of establishing one’s own family. The current study investigates these potential shifts in perception, drawing on two cross-sectional samples collected during the first year of the pandemic (January 2021; Study 1) and 2 years after the onset of the pandemic, as individuals adapted to ongoing challenges (August 2022; Study 2).

In historical context, marriage and childbirth have been cornerstones of American adulthood (Coontz, 2005). Erikson’s (1968) psychosocial model reinforced this, situating these events at the heart of adult development. However, data spanning recent decades has shown an apparent decline in the undertaking of these milestones (U.S. Census Bureau, 2021). Shifting gender roles (Gerson, 2009) have redefined domestic spheres, enabling more fluidity in decisions around marriage and childbearing. Cohabitation, premarital parenting, and voluntary childlessness are becoming more commonplace, influenced by a cultural shift toward individualism and self-expression (Finkel et al., 2014). Consequently, the personal importance placed on marriage and parenthood may have also declined, signaling a need for a reimagined view of adulthood that accommodates a greater diversity of personal trajectories.

As the COVID-19 pandemic disrupted lives, the uncertainty may have prompted many to question their life choices and their imagined future trajectories, including the desire to marry and have children. Although some studies found no impact on women’s desires to have children (Ghaffari et al., 2024), existing research from around the world has demonstrated that, amidst the early pandemic, women’s plans to have children were significantly affected. For example, women displayed heightened concern for preventing unintended pregnancies (Sen et al., 2024), and as many as 40% reported they had changed their plans to conceive (Lindberg et al., 2020). In European countries heavily impacted by COVID-19, family planning was disrupted, with many individuals reporting a decreased likelihood of having children compared to those in less affected regions (Luppi et al., 2020). A similar pattern was echoed by another Turkish study: nearly half of participants reported wanting to have a child prior to the pandemic, while only 19.2% reported wanting to have a child during the pandemic (Kaya Odabaş et al., 2024). These contradictory findings, the relatively limited research on explicit attitudes toward the complex nature of family planning—encompassing both childbearing and marriage—and the specific focus on how the pandemic affected women’s desires around family planning while largely omitting men’s perceptions underscore the need for continued research in this domain.

Beyond the COVID-19 pandemic itself, economic downturns likely heightened these doubts, leading some to question the feasibility of such commitments (Alon et al., 2020). Yet, as the pandemic continued and people adjusted to new norms, these initial apprehensions may have given way to a renewed desire for marriage and having one’s own family. Although the timing around marriage has shifted in the United States, research shows that American adults still view married life as bringing about a sense of security and stability (Gesselman et al., 2019)—a context that may have been especially appealing as the urgency of the pandemic began to decrease.

The theoretical frameworks of Social Exchange Theory (Homans, 1958; Thibault and Kelley, 1959) and Crisis Theory (Caplan, 1989; Hobbs, 1984; Turner and Avison, 1992) provide the foundations for understanding these shifts in the personal importance of marriage and having children. The former posits that relationships are a function of their perceived costs and benefits, a ratio that the pandemic likely skewed substantially. As the pandemic introduced global economic uncertainties and lifestyle disruptions, the perceived costs of entering into relationships that could lead to marriage, or of having children, could be perceived as higher than in previous non-pandemic years. Meanwhile, Crisis Theory suggests that in times of upheaval, individuals reassess their values, potentially leading to a recalibrated sense of what is truly meaningful in their lives (Moos and Schaefer, 1986). The prolonged nature of the COVID-19 pandemic and its wide-reaching impacts could have compelled many individuals to try and make meaning, form social connections, and attempt to create more stability. As marriage and having one’s own family are conventional markers of stability, it follows that these milestones may have been reconsidered as sources of personal meaning and security. As such, the personal importance of marriage and having children may have shifted during the pandemic, but in a nuanced fashion reflecting the immediate impact of and subsequent adjustment to the pandemic. Individuals may have initially felt that these goals were less important than they had previously been, due to the tumultuousness of the early pandemic context; however, as the pandemic continued, marriage and having children may have re-emerged as personally important milestones as individuals sought to establish more connection, stability, and support.

The present research is a secondary analysis of two cross-sectional studies of romantically unpartnered (i.e., single) and child-free U.S. adults: Study 1 was conducted in the first year of the COVID-19 pandemic (January 2021), and Study 2 was conducted in the second year (August 2022). The research questions posted are as follows:

1. Did the self-reported personal importance of marriage shift in comparison to how participants felt the year prior to the pandemic?
2. Did the self-reported personal importance of having shift in comparison to how participants felt the year prior to the pandemic?
3. Which sociodemographic factors correlate with these potential shifts in the valuation of marriage and having children?

## 2 Study 1 materials and methods

### 2.1 Data collection

Data were drawn from a larger study conducted in January 2021 that assessed people’s romantic and sexual lives during the pandemic. The nationally representative sample included 2,000 American adults, both singles and people in relationships. Participants were required to be at least 18 years old, proficient in the English language, and living in the United States. The study was approved by the Institutional Review Board at [blinded for review]. Participants were recruited by Prodege^®^, using independent opt-in Internet research panels for a population-based cross-sectional survey. Panelists are drawn from a diverse pool of established participants who have been recruited over several years from mailings, referrals, corporate partnerships, and internet recruitment. Recruitment targeting was based on demographic distributions (i.e., age, gender, ethnicity, region, income) to match the demographics reflected in the most recent U.S. Census. However, note that participant age was restricted to between 18 and 45 years at the request of the study’s funder, Hearst Communications. No *a priori* power analyses were performed; instead, we planned to recruit 2,000 participants to maximize the sample size in light of the amount of funding received. To be included in the current analyses, participants had to report that they were at least 18 years old, were single, had no children, and identified as a man or woman; those not meeting these criteria were excluded.

### 2.2 Participants

Study 1 had 513 participants (*M*_age_ = 28.67, SD = 7.80, range: 18–45); 56% were men, 44% were women. Most were heterosexual (87%), White (54%), and employed (54.2%). See Table 1 for full demographics.

**Table 1:** Participant demographics.

	Study 1 participants (*n* = 513)	Study 2 participants (*n* = 1,850)
Age (years)		
Mean (SD)	28.67 (7.80)	28.42 (7.93)
Gender (%)		
Man	287 (55.9%)	952 (51.5%)
Woman	226 (44.1%)	898 (48.5%)
Sexual orientation (%)		
Heterosexual	444 (86.5%)	1,520 (82.2%)
Lesbian/Gay/Bisexual	69 (13.5%)	300 (16.2%)
Race/ethnicity (%)		
Black/African American	90 (17.5%)	445 (24.1%)
East Asian	34 (6.6%)	114 (6.2%)
Hispanic or Latino	89 (17.3%)	308 (16.6%)
North American Indian or Pacific Islander	3 (0.6%)	36 (1.9%)
South Asian	8 (1.6%)	44 (2.4%)
White	277 (54%)	1,074 (58.1%)
Other	12 (2.3%)	16 (0.9%)
Yearly household income (%)		
Less than $15,000	128 (25.0%)	354 (19.1%)
$15,000 to $29,999	115 (22.4%)	321 (17.4%)
$30,000 to $44,999	91 (17.7%)	333 (18.0%)
$45,000 to $59,999	64 (12.5%)	260 (14.1%)
$60,000 to $74,999	44 (8.6%)	175 (9.5%)
$75,000 to $99,999	33 (6.4%)	212 (11.5%)
$100,000 to $149,999	22 (4.3%)	152 (8.2%)
$150,000 or more	16 (3.1%)	43 (2.3%)
Employment status (%)		
Employed	278 (54.2%)	1,197 (64.7%)
Unemployed	145 (28.3%)	340 (18.4%)
Other (e.g., retired, student)	90 (17.5%)	313 (16.9%)

### 2.3 Measures

#### 2.3.1 Demographics

Participants self-reported age, gender identity, sexual orientation, race/ethnicity, household income in 2020, and employment status.

#### 2.3.2 Importance of marriage and having children

Participants responded to the question “Compared to how you felt in the year before the pandemic, have your feelings changed about any of the following?,” with “Marriage” and “Having children” as two of a small group of items displayed. Participants responded on 7-point Likert scales where 1 = *much less important*, 4 = *the same*, and 7 = *much more important.* See Table 2 for complete means and standard deviations for all demographic variables of interest.

**Table 2:** Descriptive statistics for Studies 1 and 2 measures.

Variable	*N*	Scale	Mean	SD
Study 1	513			
Feelings toward marriage	–	1–7	4.37	1.86
Feelings toward having children	–	1–7	3.93	1.87
Study 2	1,850			
Feelings toward marriage	–	1–5	2.9	0.86
Feelings toward having children	–	0–1	0.59	0.49

### 2.4 Study 1 analysis plan

To determine whether self-reported attitudes toward the importance of marriage or having children have changed due to the COVID-19 pandemic, we conducted two one-sample *t*-tests comparing the sample averages with the midpoint of the response scales. The midpoint of the scale (4) was labeled “the same,” which reflects no change in attitudes regarding personal importance of marriage or having children. A non-significant *t*-test result would correspond to no general change in attitudes toward the importance of marriage/having children compared to attitudes in the year prior to the pandemic.

Next, we examined several demographic subgroups to assess if certain groups expressed shifts in their attitudes toward the importance of marriage or having children compared to how they felt in the year prior. We conducted two linear regressions with the importance of marriage as the outcome variable in model 1, and importance of having children as the outcome variable in model 2. Our predictor variables in both models included: age (mean-centered), gender (female = 0, male = 1), sexual orientation (lesbian / gay / bisexual [LGB] = 0, heterosexual = 1), and income was entered into the model as a covariate. Note that no *a priori* power analyses were conducted, but a post-hoc power analysis suggested that 513 participants had 99% power to detect a small effect size (*f* ^2^ = 0.02) in a linear regression with six categorical predictors and two continuous predictors.

## 3 Study 1 results

### 3.1 Self-reported shifts in the importance of marriage and having children

One-sample *t*-tests revealed that participants felt that marriage was less important at the time of the survey compared to how they felt prior to the pandemic, with a small effect size (*M* = 3.73, SD = 1.75; *t*(512) = −3.53, *p* < 0.001; *d* = −0.15). Examination of descriptive statistics showed that 28.7% of the sample reported that marriage was less important than it had previously been (i.e., below the 4.0 midpoint), 48.9% selected the midpoint of “*the same*,” and 22.5% reported that marriage was more important than previously (i.e., above the 4.0 midpoint).

Across the sample, participants also felt that having children was less important at the time of the survey compared to how they felt prior to the pandemic, with a moderate effect size (*M* = 3.43, SD = 1.74; *t*(512) = −7.35, *p* < 0.001, *d* = −0.33). Descriptive statistics showed that 34.9% reported that having children was less important than it had previously been, 47.8% selected ‘*the same*’, and 17.3% reported that having children had become more important than previously felt.

### 3.2 Demographic subgroups and trends in importance of marriage

Both gender (*p* = 0.006) and the covariate income (*p* = 0.02) emerged as significant predictors of self-reported shifts in the importance of marriage, *F* (4, 508) = 3.30, *p* = 0.01. Women (vs. men) and participants with higher (vs. lower) income were more likely to feel that marriage had grown in importance to them. There were no effects for sexual orientation or age. See Table 3 for all regression coefficients.

**Table 3:** Regression coefficients for Studies 1 and 2.

Outcome	Predictor	*B*	*SE*	β	*p*	95% CI_B_
Study 1						
Importance of marriage^a^	Age	0.000	0.010	−0.001	0.984	−0.020, 0.019
	Gender	−0.428	0.156	−0.121	0.006	−0.733, −0.122
	Sexual orientation	−0.060	0.225	−0.12	0.788	−0.502, 0.381
	Income	0.096	0.040	0.106	0.016	0.018, 0.174
Importance of children^b^	Age	0.003	0.010	0.012	0.785	−0.017, 0.022
	Gender	−0.260	0.155	−0.074	0.095	−0.565, 0.046
	Sexual orientation	0.332	0.225	0.065	0.140	−0.109, 0.773
	Income	0.077	0.040	0.085	0.054	−0.001, 0.155
Study 2						
Importance of marriage^c^	Age	0.001	0.003	0.009	0.704	−0.004, 0.006
	Gender	−0.002	0.042	−0.001	0.963	−0.085, 0.081
	Sexual orientation	0.048	0.057	0.020	0.399	−0.064, 0.160
	Income	0.025	0.010	0.057	0.016	0.005, 0.046
Importance of children^d^	Age	0.002	0.001	0.039	0.098	0.098, 0.000
	Gender	0.039	0.022	0.041	0.081	−0.005, 0.083
	Sexual orientation	0.091	0.030	0.071	0.003	0.032, 0.151
	Income	−0.007	0.006	−0.029	0.213	−0.018, 0.04

a *R*^2^ = 0.03, *R*^2^ adjusted = 0.18, *F*(4, 508) = 3.30, *p* = 0.011.

b *R*^2^ = 0.02, *R*^2^ adjusted = 0.01, *F*(4, 508) = 2.14, *p* = 0.074.

c *R*^2^ = 0.004, *R*^2^ adjusted = 0.002, *F*(4, 1815) = 1.72, *p* = 0.142.

d *R*^2^ = 0.10, *R*^2^ adjusted = 0.01, *F*(4, 1815) = 4.511, *p* = 0.001.

### 3.3 Demographic subgroups and trends in importance of having children

Results of the linear regression indicated that the covariate income (*p* = 0.05) was a significant predictor of the importance of having children; participants with higher income were more likely to report that having children had grown in importance to them. There were no other significant demographic differences in self-perceived importance of having children.

## 4 Study 1 summary

Study 1 investigated self-reported changes in the importance placed on marriage and childbearing among single, childless U.S. adults during the first year of the pandemic, compared to how they felt in the prior year. Findings suggest that both childbearing and marriage decreased in importance overall but grew in importance for people with higher incomes and for women (marriage only).

## 5 Study 2 methods and measures

### 5.1 Data collection

Data were drawn from a larger study conducted in August and September of 2022 as part of the Singles in America (SIA) study. SIA is an annual cross-sectional survey on the attitudes and behaviors of a representative sample of single people in the United States. Study 2 was funded by the Match Group, which operates Match.com and other online dating platforms. No participants were drawn from the Match population or any subsidiary sites, nor were any questions associated with Match.com content. No participants were screened for engagement with any Match services.

Participants were recruited exclusively by Dynata (Dallas, TX, United States), using independent opt-in Internet research panels for population-based cross-sectional surveys. Panelists were drawn from a pool of established participants who have been recruited over several years from a variety of venues, including paper and electronic mailings, referrals, corporate partnerships, and internet recruitment. Participants were recruited from these opt-in research panels, with recruitment targeting based on demographic distributions (i.e., age, gender, ethnicity, region, income) reflected in the most recent U.S. Census.

SIA inclusion criteria required being at least 18 years old, being fluent in English, and having a relationship status of single (i.e., unmarried and single, defined as not in a committed relationship). Research panelists were required to verify their identity through a certification process, which employs validation technologies in real-time to identify and screen out duplicate and inattentive respondents that may attempt to take a survey. The study was approved by the Institutional Review Board at [blinded for review]. No *a priori* power analyses were conducted; the annual SIA survey seeks to recruit 5,000 participants per year. To be included in the analyses, participants had to report (1) they were at least 18 years old, (2) they were between the ages of 18 and 45 (to replicate the age distribution in Study 1) (3) were single, (4) reported having no children, and (5) identified as a man or a woman; participants not meeting these criteria were excluded from analyses.

### 5.2 Participants

Study 2 had 1,850 participants (*M*_age_ = 28.42, *SD* = 7.93, range: 18–45). Of the 1,850 participants, 52% identified as men and 48% as women. Most were heterosexual (82.2%), White (58.1%), and employed (64.7%). See Table 1 for complete participant demographics.

### 5.3 Measures

#### 5.3.1 Demographics

Participants self-reported their demographics including age, gender identity, sexual orientation, race / ethnicity, approximate income in 2021, and employment status.

#### 5.3.2 Importance of marriage and having children

Participants were asked, “Since 2019 (before the pandemic), have your feelings about marriage changed?” and were shown the question stem “I want to get married…” followed by a 5-point Likert scale where 1 = *a lot less now*, 3 = *the same as before*, and 5 = *a lot more now*. Similarly, participants were asked, “Did the COVID-19 pandemic change your feelings about having children?” Participants responded on a three point Likert scale where 1 = *yes, it made me want them less*, 2 = *no*, and 3 = *yes, it made me want them more*.

### 5.4 Study 2 analysis plan

The same analyses were conducted as in Study 1, including the one-sample *t*-tests and linear regressions to determine sociodemographic differences in self-reported importance of marriage and having children. Note that no *a priori* power analyses were conducted, but a post-hoc power analysis suggested that 1,850 participants had 99% power to detect a small effect size (*f* ^2^ = 0.02) in a linear regression with six categorical predictors and two continuous predictors.

## 6 Study 2 results

### 6.1 Reported changes in the importance of marriage and having children

Overall, participants reported that they wanted to get married just as much as they did in 2019, before the COVID-19 pandemic—the mean of 2.98 (SD = 0.90) did not differ from the 3.00 midpoint indicating they felt “the same as before” (*t*(1,849) = −0.96, *p* = 0.34). Descriptive statistics showed that 18.6% of the sample reported that marriage was less important than it had been in 2019 (i.e., below the 3.0 midpoint), 63.6% selected the midpoint of “*the same*,” and 17.8% reported that marriage was more important than previously (i.e., above the 3.0 midpoint). See Table 2 for complete means and standard deviations for all demographic variables of interest.

Conversely, participants reported a change in their desire to have children compared to how they felt prior to the pandemic in 2019. The sample mean significantly differed from the scale midpoint of 2.0 in a one-sample *t*-test (*M* = 1.93, SD = 0.48; *t*(1,850) = −6.45, *p* < 0.001). Most (76.9%) participants reported no change in the importance of having children, but 15.1% reported that they now wanted to have children less; 8% now wanted to have children more than before the pandemic.

### 6.2 Demographic subgroups and trends in the importance of marriage

Results of the linear regression indicated that the covariate income (*p* = 0.02) was a significant predictor of the importance of getting married; participants with higher income were more likely to report that getting married had grown in importance to them. There were no effects for sexual orientation, gender, or age.

### 6.3 Demographic subgroups and trends in the importance of having children

Sexual orientation was a significant predictor of the importance of having children: heterosexual people were more likely to report that having children had grown in importance to them relative to people who identified as LGB. There were no effects for age, gender, or income.

### 6.4 Study 2 summary

Study 2 aimed to revisit Study 1’s findings regarding how single, childless U.S. adults value marriage and children during the ongoing COVID-19 pandemic. Contrary to Study 1, this later assessment found no difference in the self-reported importance of marriage at the time of the survey—August 2022—compared to how participants felt in 2019, prior to the pandemic. Study 2 did not replicate Study 1’s findings regarding income and gender, but found differences by sexual orientation, with heterosexual people reporting more of an increase in importance around having children post-pandemic.

## 7 Discussion

The overarching goal of the current research was to explore shifts in perceptions surrounding the importance of marriage and having children in the context of the COVID-19 pandemic. In line with the premises of social exchange theory (Cropanzano and Mitchell, 2005) and crisis theory (Turner and Avison, 1992), our findings from two cross-sectional studies suggest that some individuals engaged in a re-evaluation of their personal priorities, but this reassessment has manifested in nuanced ways over time.

The results from Study 1 indicated during the first year of the pandemic, the importance of marriage and having children showed a general decline in self-reported importance among single, childless U.S. adults. This supports the notion, central to Social Exchange Theory, that the perceived costs of marriage and parenting rose during the early pandemic—perhaps due to economic uncertainties or the increased complexities of social interactions. Nonetheless, for higher-income individuals and women, the pandemic seemed to amplify the importance of marriage as a milestone. This divergence may reflect an economic buffer that allows for the maintenance or even enhancement of personal goals during tumultuous times (Carroll et al., 2020; Edin and Reed, 2005), or it could point to the pursuit of marriage as a form of stability in an unstable world (Gesselman et al., 2019)—a reflection of Erikson’s (1968) theory of psychosocial development, which situates intimate relationships as a core aspect of adulthood.

In contrast, Study 2’s findings from the second year of the pandemic depict a stabilization of attitudes toward marriage and childbearing, which realigns with pre-pandemic levels. The near-absence of demographic predictors, unlike in Study 1, could be indicative of a collective adaptation to the ‘new normal’, where the initial shock and recalibration have settled into a reinstated value system that once again embraces the social ideals of marriage and starting one’s own family through childbearing. This is consistent with Crisis Theory (Turner and Avison, 1992), which posits that following a period of disarray, individuals will strive to find meaning and return to a state of equilibrium.

Notably, Study 2 also revealed that sexual orientation emerged as a significant factor, with heterosexual participants reporting an increased valuation of having children post-pandemic. This finding may be reflective of norms around heterosexual relationships and the expectation that heterosexual couples will eventually reproduce—a norm that does not translate to cisgender gay or lesbian couples, or highlights significant barriers in accessing fertility treatment, perhaps making this experience less accessible and salient (DiLapi, 1989; Friedman, 2007; Kirubarajan et al., 2021; Lehavot et al., 2009).

It is important to note that factors in addition to the COVID-19 pandemic may have influenced the attitudes associated with the importance of marriage and having children during Study 1 (January 2021). Specifically, the early, pre-vaccine portion of COVID-19 was associated with an economic downturn in the United States, which led to financial struggles for many Americans (Parker et al., 2020) and workforce limitations for parents who were without childcare due to closures (Kochhar, 2020). Due to these factors, many Americans may have temporarily adjusted attitudes toward the importance of marriage and children beyond the health-threats of the COVID-19 pandemic. As the economy began to reopen and optimize, attitudes associated with family matters may have similarly experienced a rebalancing of preferences. Based on the cross-sectional nature of the present research, only associations can be suggested; future work should assess causal relationships in the influence of these domains, as listed and beyond, such as in assessing the role of mental health on influencing attitudes surrounding marriage and children.

Nonetheless, these findings suggest a trajectory of initial disruption and subsequent recalibration of values regarding marriage and childbearing among U.S. adults without children. This trajectory is a testament to the human capacity for resilience and the search for stability through traditional social constructs, even as the broader societal narrative around these institutions continues to evolve. The varying impact of the pandemic across different demographic groups (i.e., income, gender, sexual orientation) also underscores the need to consider a multiplicity of experiences when examining the effects of societal crises on personal values.

Furthermore, the present research sought to cultivate a deeper understanding of how single, childless adults responded to the COVID-19 pandemic through the lens of key life-course milestones. In recent decades, birth rates in the United States have declined (U.S. Census Bureau, 2021). Understanding factors that may persuade single adults toward—or away from—family building is crucial for understanding and anticipating future trends, informing social support, and understanding the evolving landscape of romantic relationships and parenthood.

As Cherlin (2010) noted, marriage has transitioned from a fundamental step to an aspirational one for many individuals. The COVID-19 pandemic has, in some ways, accelerated this perspective, bringing into sharp relief the choices surrounding personal milestones. In doing so, the pandemic has prompted a deeper interrogation of what these traditional markers of maturity mean in the context of contemporary adulthood. The present research contributes to the burgeoning literature on how macro-level disruptions influence micro-level decisions and values (e.g., Cohan and Cole, 2002). It underlines the importance of considering temporal dynamics when assessing the impact of crises, as immediate effects may give way to different outcomes with the passage of time. As society continues to navigate the long-term implications of the COVID-19 pandemic, these findings offer a lens through which to view the ongoing reconfiguration of personal and societal expectations of adulthood.

### 7.1 Limitations and future directions

Although this research had many strengths, there are some limitations and avenues for future research to aid in the understanding of future plans for marriage and children. The present research relied upon single-item measures, adapted by the research team and those involved with the larger SIA survey from other published research, to assess the importance of marriage and having children in each study. Recent research associated with single-item measures, particularly in the realm of psychological research, demonstrates validity and reliability that are often comparable to measures with multiple items (e.g., Ahmad et al., 2014; Ang and Eisend, 2018) and can adequately capture concepts like relationship satisfaction (Fülöp et al., 2020) and loneliness (Reinwarth et al., 2023). As these items were taken from broader studies, single-item measures were used because they have been found to be particularly useful in minimizing participant fatigue and increasing completion rates (Allen et al., 2022; Sloan et al., 2002; Wanous et al., 1997). However, future research should aim to use more robust, psychometrically validated quantitative measures and include open-ended qualitative items to develop a more comprehensive understanding regarding attitudes surrounding the importance of marriage and having children, particularly as it relates to the impact of the COVID-19 pandemic.

Additionally, the two studies included in this research employed large-scale survey sampling with the aim to collect a representative sample of adults in the United States. However, it is important to note that both surveys were conducted online; as such, a subgroup of the U.S. population—those with no access, or unstable access, to the Internet—are likely to be excluded from the present datasets, as they could not be recruited via online methods. This excluded group of individuals likely includes many who have low income or unstable housing, both of which could have been exacerbated by the COVID-19 pandemic and could affect desires for marriage and having children. Future studies should employ stronger strategies for more comprehensive sampling.

Finally, both of the studies were cross-sectional in nature. Although this provides snapshots into 2021 and 2022, the effects of the pandemic, particularly pertaining to marital and family life, may have been shorter term or may come to light at a later date. Being able to conduct a longitudinal study that includes future timepoints would be helpful to determine if these effects will re-emerge in later years. This would be helpful in determining if there are residual effects throughout a longer period of time and how different impacts from the pandemic could influence individuals’ behaviors and decisions related to marriage and childbirth, who were younger than the surveyed samples.

### 7.2 Conclusion

The current cross-sectional research offers a nuanced illustration of how the COVID-19 pandemic has affected the evolving cultural narrative around marriage and childbearing in the United States. Amidst the global crisis, there was an initial decline in self-reported importance of these traditional milestones among single, childless adults (Study 1), with a subsequent return to pre-pandemic attitudes as individuals adapted over time (Study 2). This pattern reflects a complex interplay between societal expectations, personal aspirations, and the resilience of traditional values in the face of unprecedented change. These findings provide evidence for the adaptability of human values in the context of crisis and suggest a dynamic, rather than static, re-evaluation of the roles that marriage and parenthood play in the lives of modern adults. This research enriches our understanding of the social dynamics underpinning American life and highlights the enduring significance of these institutions, even as their place in the timeline of adulthood continues to be negotiated.

## Data Availability

The raw data supporting the conclusions of this article will be made available by the authors, without undue reservation.
